# Customized Borosilicate Bioglass Scaffolds With Excellent Biodegradation and Osteogenesis for Mandible Reconstruction

**DOI:** 10.3389/fbioe.2020.610284

**Published:** 2020-12-18

**Authors:** Peng Zhang, Kang Yang, Ziyu Zhou, Xingrong Zhu, Wenchao Li, Chuanliang Cao, Kui Zhou, Lan Liao, Fanrong Ai

**Affiliations:** ^1^School of Stomatology, Nanchang University, Nanchang, China; ^2^The Key Laboratory of Oral Biomedicine, Jiangxi Province, Nanchang, China; ^3^School of Pharmacy, Nanchang University, Nanchang, China; ^4^School of Mechatronics Engineering, Nanchang University, Nanchang, China; ^5^Nanchang Municipal Key Laboratory of 3D Bioprinting Technology and Equipment, Nanchang, China

**Keywords:** borosilicate bioglass, mandible reconstruction, degradation, osteogenesis, 3D printing

## Abstract

Graft reconstruction of the mandible is an important approach that aims at improving the appearance and functionality of defected mandibles. The traditional implant materials are generally bioinert, non-degradable, and that they lack favorable pore structures for cell proliferation, which limit their clinical application. In this study, we used boron-containing bioactive glass which was combined with a three-dimensional (3D) printing technology to construct an osteoinductive implant scaffold, according to the imaging instructions of CT scan on bone defects. Here, the boron-containing bioglass scaffold (B-BGs) was prepared through sol-gel processing and a 3D print technique. Different boron content of borosilicate bioglass was prepared by incorporating B_2_O_3_ (molar: 19.4 and 38.8%) into 58S bioglass to replace parts of SiO_2_. For fabricated mandible implants through three-dimensional 3D printing of B-BGs (size: 8 × 2 mm; pore size: 250 μm) modified with borosilicate bioglass powder and sodium alginate. Notably, the compressive strength of the B-BGs was about 3.8 Mpa, which supported mandibular activity. Subsequently, the excellent biocompatibility of B-BGs was confirmed using cytotoxicity *in vitro* studies. Finally, data from *in vivo* experiments demonstrated that the B-BGs could promote bone regeneration and they could almost get completely degraded within 4 weeks. Our results showed that the boron-containing bioglass could repair mandibular defects.

## Introduction

Mandibular defects usually occur after bone tumor resections, osteomyelitis, trauma, and congenital deformities and are accompanied by tissue destruction and alveolar bone absorption, causing insufficient bone mass (Khan et al., 2015; Brown et al., 2016; Beth-Tasdogan et al., 2017). Regardless of their etiology, they can cause severe alteration on the mandibular contour, which could also impact its morphology, and function. Surgical reconstruction of segmental mandibular remains a significant clinical problem because of the limited self-repair capacity of the mandibles (Khan et al., 2015; Nickel et al., 2018; Sculean et al., 2019). Clinically, to promote the healing of fractures and large defects autologous or allogeneic bone transplantation methods are still the most popular options (Myeroff and Archdeacon, 2011; Fillingham and Jacobs, 2016). However, the use of these materials also imposes many limitations. First, the use of the allogeneic bone transplantation method could trigger immune reactions and possible disease transmissions, which is challenging for patients diagnosed with tumors or are suffering from severe infections (O'Sullivan et al., 2017). Secondly, due to donor limitations, it is often challenging to obtain a graft with a compatible shape and size to the damaged site. However, some inert implant materials, like titanium alloy materials, seem to avoid these risks. Nevertheless, the overall implant success rate of titanium and its alloy is only 93.0–96.6% after 6 months, which is inevitably accompanied by the possibility of implant-related complications such as screw loosening and steel plate breakage (Mestas et al., 2016; Bormann et al., 2018). These disadvantages result in the traditional inert materials being nearly difficult to reconstruct well in the defect (Namm et al., 2018; Ramadanov et al., 2020).

It is therefore deemed critical that a more effective treatment strategy to fast restore the function and morphology of the mandible is required. The ideal mandibular repair material requires macropore and micropore structures that are similar to the bone to support vascular endogenesis, proliferation of the cell, regeneration of the bone, and cytokine exchange, and secondly, it should be compatible with the defect to support mandibular activity (Kim et al., 2017; Zhang et al., 2019; Chen et al., 2020). The three-dimensional printing technology has emerged as a promising modality with considerable advantages in customizing the implants to meet diverse individual needs. This technique has been used in bone and joint repair, and vascular reconstruction (Lim, 2017; Shi et al., 2017; Pan et al., 2020). In our study, an improved bioactive glass combined with a 3D printing technique was used in the construction of an osteoinductive implant scaffold. Here, we first used a biocompatible sodium alginate solution as a solvent to dissolve the toxicity and irritation caused by the residue of traditional organic solvents and accomplish rapid prototyping ability through adjusting the different sodium alginate and bioglass ratios. Subsequently, the 3D printing technique used CT scan data from the defect sites, to generate B-BGs that mimic the structure and shape these sites.

Although tooth loss is usually attributed to trauma, tumors, etc. it is usually accompanied by the destruction and absorption of alveolar bone, which results in insufficient bone mass, particularly in the upper anterior teeth region. This makes it difficult for the traditional inert materials to reconstruct well in the defect. Different studies have suggested a series of promoting materials that have osteoinduction and vascular regeneration properties. For instance, bioglass has osteoinductive properties, and its ionic products may prompt osteoblast activity, which is deliberated to be caused through the over-expression of osteoblast-related genes present in it. Other materials like VEGF, PuF, etc. promote an increase in bone mass. Also, this bioglass promotes the proliferation of cells through IGF and glutamic acid synthesis. As a trace element, boron (B) has been considered essential for bone physiology. It is playing a regulatory role in the metabolism of various micronutrients such as calcium, phosphorus, aluminum and molybdenum. Moreover, it is reported that incorporation of B in bioactive glass could boost osteogenesis *in vitro* and *in vivo*. Therefore, it has received massive attention and is used in bone defect replacement repair caused by tumors and osteoporosis (Hoppe et al., 2011; Zeimaran et al., 2015).

In this study, a B-BG was obtained from boron which completely replaced sodium. Studies from previous articles have confirmed its osteoinductive aptitude and degradation characteristics (Xia et al., 2019; Deilmann et al., 2020; Houaoui et al., 2020; Li et al., 2020). Also, implants that were hard to degrade were described to cause long-term inflammatory reactions, and stress shielding occurs, which results in bone resorption and implant loosening due to mechanical properties that do not match that of the natural bone (Fedorowicz et al., 2007; Xu et al., 2020). However, the degradable bioactive glass can avoid the stress shielding effect and offer a place for new growth of bone as the material degrades. Notably, a good degradable implant must have a degradation rate that matches that of bone repair (Han et al., 2016; Yang et al., 2019). Reports have indicated that the traditional 58S bioglass, can only degrade 8% within 28 days, whereas the time taken by the conventional mandible repair technique is only 4–6 weeks, hence it inhibits the regeneration of the bone during its late-stage (Bak et al., 2010; Wong et al., 2010; Shuai et al., 2016). Nevertheless, the slow rate in degradation of the 58S bioglass could be as a result of the high dense mineral layer. For it to accelerate the degradation rate, studies have shown that the use of boron instead of silicon in the bioglass helps (Sanz-Herrera and Boccaccini, 2011; Moonesi Rad et al., 2019). With the introduction of the seed-soil theory, studies have also considered that angiogenesis is a vital guarantee for bone reconstruction and even long-term survival of implants. The osteoinduction effect of the boron-doped bioglass has been reported extensively, and some recent articles have shown that it can promote vascular remodeling (Westhauser et al., 2019). In our experiment, the boron-doped bioglass was used and its degradation rate and bioactivity improved for better mandibular repair.

## Materials and Methods

### Synthesis of Boron-Contain Bioactive Glass

First, the B-BG was synthesized according to our previously developed methods (Ai et al., 2020). Then, ethanol was briefly dissolved in deionized water under constant magnetic stirring followed by the addition of the respective amounts of the individual precursors. Notably, the precursors were added as stated in the order below and each precursor was ensured that it completely dissolved before the next one was added. Tetraethyl orthosilicate (TEO, Aladdin, Shanghai, China), calcium nitrate tetrahydrate (Aladdin, Shanghai, China), triethyl phosphate (TEP, Aladdin, Shanghai, China), and Trinbutyl Borate (TBB, Aladdin, Tianjin, China) were used as precursors for SiO_2_, CaO, P_2_O_5_, and B_2_O_3_, respectively. Subsequently, nitric acid (Macklin, Shanghai, China) was added to catalyze the hydrolysis reaction and the resulting solution stirred for 24 h at room temperature. Next, this solution was aged and dried for 48 h at a temperature of 80°C. The resulting gel was dried and calcinated for 2 h at a temperature of 800°C and a heating rate of 1°C min−1 and then naturally cooled in the furnace. Table 1 illustrates the different compositions that were used in the preparation of Boron-containing Bioactive glass samples such as 0B, 1B, and 2B. The calcined product was ground in a mortar and pestle to obtain a fine powder which was sieved through a 200 mesh (75 μm).

**Table 1:** The weight percentage (wt.%) of Boron-containing bioglass composition.

**Batch**	**58S-0B**	**58S-1B**	**58S-2B**
**Weight percentage (wt.%)**			
SiO_2_	58.2	38.8	19.4
CaO	32.6	32.6	32.6
P_2_O_5_	9.2	9.2	9.2
B_2_O_3_	0	19.4	38.8

### Fabrication and Characterization of 3D Printed Scaffolds

To prepare ink for 3D printing, B-BG powders and 30 wt% sodium alginate (SA, Macklin, Shanghai, China) were mixed to obtain an aqueous solution, which was stirred thoroughly to get a well-mixed composite mixture. Here, the scaffolds were produced using an Inkjet 3D Printer. Next, the ink was printed through a nozzle using a layer-by-layer method. Notably, the printing extrusion speed set at 0.007 mm/s, and layer height and wire spacing set at 0.45 and 1.2 mm, respectively. Consequently, the prepared bioglass scaffolds were dried at room temperature and sintered at 800°C for 6 h.

Then, the selection of scaffold samples with a diameter of 8 mm and a height of 2 mm was done to test different mechanical properties. Afterward, the stress-strain curve of the B-BGs and that of the hydroxyapatite scaffold were measured using a Universal Testing Machine (SUNS, CMT560503050100, Shenzhen, China), and the compressive strength was calculated based on the obtained stress-strain curves. Subsequently, a Scanning electron microscope (JEOL Ltd, JSM-6701F03040700, Tokyo, Japan) was used to collect the surface structure and morphology of the stent. Lastly, X-Ray diffraction (Materials Talks, Empyrean 03030502, Netherlands) was performed to characterize the composition of hydroxyapatite and bioglass scaffold crystals and the formation of crystals throughout the process of bioglass mineralization.

### *In vitro* Mineralization

The mechanism of bone-like apatite formation was investigated by soaking B-BGs in a simulated body fluid (SBF, Servicebio, Wuhan, China), in the mass ratio of 1: 100 and kept at 37°C. Next, the B-BGs were tested for mineralization after 42 days of immersion using the XRD analysis.

### *In vitro* Cell Experiments

Sprague Dawley (SD) rats used in this experiment were procured from the Jiangxi University of Traditional Chinese Medicine. All animal experiments were approved by the Ethical Committee of the Second Affiliated Hospital of Nanchang University. To perform *in-vitro* cell experiments, the rat bone marrow mesenchymal stem cells (rBMSC) were first extracted, as outlined in previous studies (Yao et al., 2019). These cells were maintained in Dulbecco's Modified Eagle's Medium (DMEM/F-12, BI, Israel) which was supplemented with 10% fetal bovine serum (FBS, BI, Israel) and 1% penicillin-streptomycin (P/S, Thermo Fisher Scientific, MA, USA). Next, the B-BGs ionic dissolution product was prepared according to the ISO10993-5 standard protocol. In a nutshell, 1 g of the scaffold was added to 5 ml of DMEM/F-12 without serum, then incubated for 24 h at 37°C, and the ionic dissolution product was collected and stored at 4°C. Subsequently, the effect of the B-BGs ionic dissolution product on cell proliferation and survival was assessed using the cck-8 assay (Beyotime, Shanghai, China). Briefly, the rBMSC were seeded in 96-well plates at an initial density of 3 × 10^3^ cells /well with different B-BGs ionic extracts concentrations following the manufacturer's recommendations. Notably, all cell cultures were performed at 37°C under a humidified atmosphere of 5% CO_2_. Subsequently, at days 1, 3, and 7, serum-free medium supplemented with 10% cck-8 was added to each well. Consequently, the cells were incubated for 2 h at 37°C, and their absorbance measured at 450 nm using a microplate reader to evaluate their cell viability. Then, we used a dead cell staining kit (Yeason, Shanghai, China) to directly observe live/dead cells. Here, we inoculated 5 × 10^3^ rBMSC in a 24-well plate, and used a diluted extract in the ratio of 1: 2 to simulate the fate of cells in close contact with the scaffold. Imaging of live/dead cells on days 1, 3, and 7 were performed using a fluorescence microscope (TE2000, Nikon, Japan) under an excitation light wavelength of 490 and 535 nm, respectively.

### *In vivo* Animal Experiments

Male New Zealand white rabbit (3.5–4.0 kg) were purchased from the Department of Animal Science of Nanchang University and used to construct a rabbit mandibular defect model which directly assesses the effect and toxicity of the stent on mandibular repair. In summary, these rabbits were first anesthetized using 10% chloral hydrate (2.5 ml/kg). Next, the submandibular area was shaved, sterilized and the rabbits laid on a sterile sheet. Their skin was cut in sections, to separate the muscle and periosteum to expose the bone surface. Subsequently, a trephine bur was used to cut a circular defect (8 × 2 mm) in the region of the exposed mandibular.

The 12 New Zealand white rabbits were divided into three treatment groups: 1B- BGs, hydroxyapatite scaffolds, and blank control. The resulting scaffold was filled into the defect and sutured. Within 1 week after the operation, antibiotics were administered to the experimental animals. After 2 and 4 weeks, the rabbits were sacrificed using an intravenous injection of pentobarbital and their lower jaws were collected.

### Imaging and Pathology Examination

The mandibular specimens were taken at the 4th and 8th weeks postoperatively, and they were scanned using a CT tomography at a voltage of 80 kVp and a current of 80 mA. After the initial calibration against known density standards, each complete mandible was scanned in a chilled dH_2_O solution. Using the MicroView 2.2 software (GE Healthcare, Milwaukee, WI), two different regions of interest (ROIs) were identified for each hemi-mandible at the anterior and posterior bone graft interfaces as follows; the coronal plane was centered at the interface between the native bone and the iliac crest bone graft, which remained identifiable after graft incorporation. From this initial position, 20 frames were splined both anteriorly and posteriorly for a total of 40 consecutive frames per ROI. Next, several rotations and cropping of non-bone space were performed to guarantee uniform data measurement. Then, bone mineral density (BMD), bone volume fraction (BVF), and tissue mineral density (TMD) metrics were obtained from each of the ROI. After bone analysis, data on the anterior and posterior ROI from each animal were averaged for subsequent statistical analysis. Consequently, to assess bone density, bone volume fraction, trabecular bone separation, and bone thickness, CT scan images were then imported into a medical image processing software (Mimics 16.0; Materialize). Subsequently, to assess bone regeneration and inflammation, the obtained specimens from the hard tissue microtome were stained using a Hematoxylin-Eosin/HE Staining Kit which was procured from Solarbio, Shanghai, China. Lastly, to obtain an objective pathological evaluation, the pathological analysis of the slices was double-blinded by three experienced pathology-related practitioners.

### Statistical Analysis

All data were expressed as mean ± standard deviation for at least three independent experiments. The differences between the control and experimental groups were compared using the Kruskal-Wallis one-way analysis of variance (ANOVA) and *t*-test to calculate statistical significance (Graphpad prism 8.0.1).

## Results and Discussion

### Characterization and *in vitro* Mineralization of B-BGs

**Figure 1:**
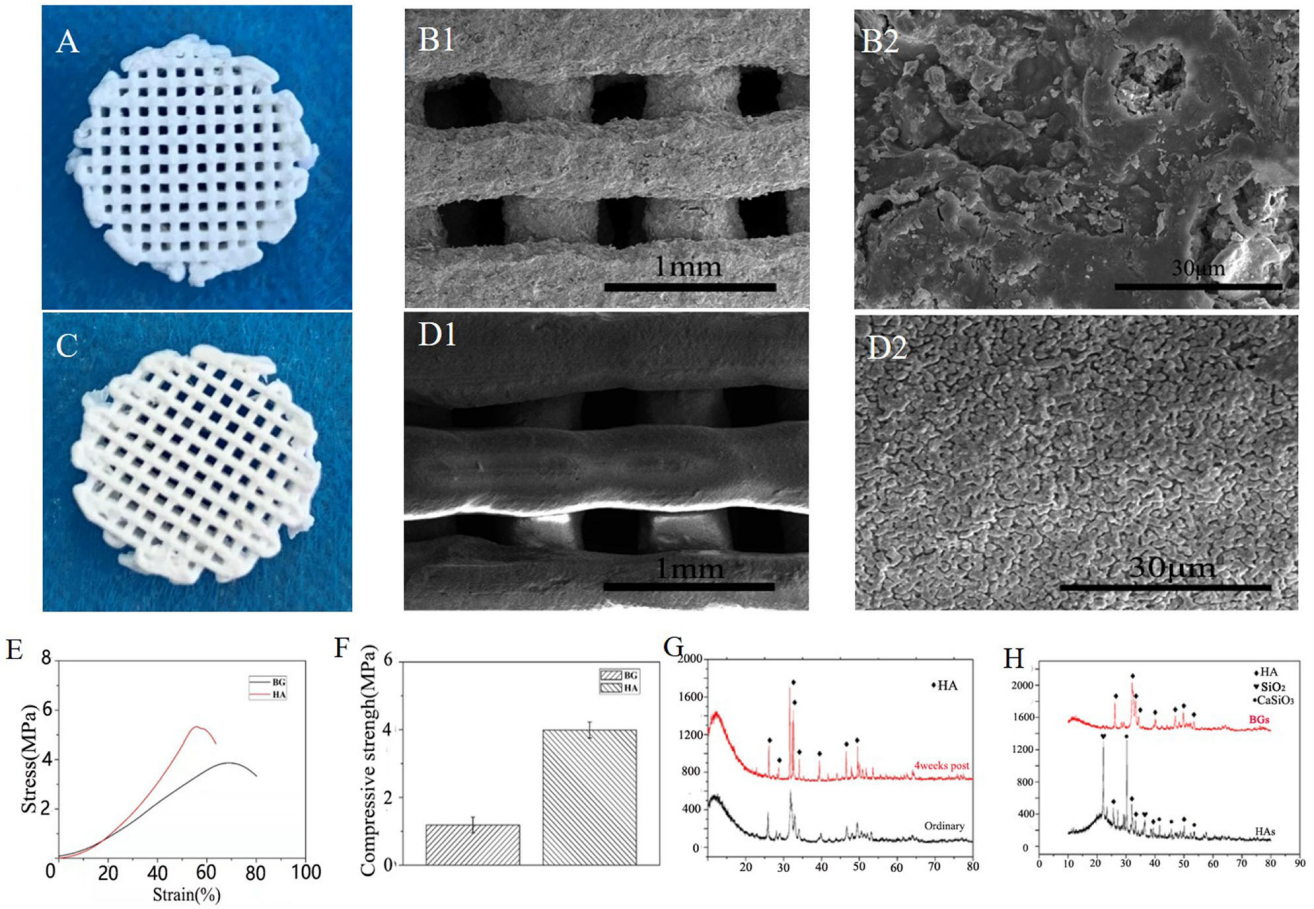
Characterization and *in vitro* degradation of the B-BGs and HAs. **(A)** Photo and **(B)** SEM images of B-BGs; **(C)** photo and **(D)** SEM images of HAs**; (E)** the stress-strain curve of the B-BGs and Has; **(F)** compressive strength of the B-BGs and HAs; **(G)** The x-ray diffraction patterns of the ordinary and 4 weeks of the B-BGs; **(H)** The x-ray diffraction patterns of the B-BGs and Has.

Hydroxyapatite, one form of calcium phosphate coating, has been widely used as a biocompatible scaffold for bioengineered bone implants (Wang et al., 2020; Yu et al., 2020; Zhu et al., 2020). However, it seems to have some certain type of acidity, whereas most reports have indicated that an alkaline environment inhibits the production of osteoclasts and promotes the proliferation of osteoblasts, thus reducing bone resorption (Arnett and Dempster, 1986; Arnett, 2008; Galow et al., 2017). On the other hand, bioglass contains alkali metals, which could slowly maintain a certain alkaline condition in the body fluid environment for a long time (El-Rashidy et al., 2017), hence, there could be a probability that it is a better material for the regeneration of bones. The SEM image analysis shows that this bioglass scaffold has a complete macrostructure, and a uniform pore size of 0.25 mm is formed through printing, which has a rough surface morphology that allows proper cell attachment. Besides, higher magnification microscopic images have shown that the pores ranged in size from several microns to tens of microns on the surface of this bioglass scaffold, whereas, as illustrated in Figures 1A–D, the hydroxyapatite scaffold only had a uniform pore size of about 1 μm which was observed on its surface. Our study hypothesized that this difference could be attributed to the volatilization of the solvent and the non-linear shrinkage of the bioglass, compared to the formation of crystals during the calcination of the hydroxyapatite. Moreover, the hypothesis that hydroxyapatite mainly exists in the form of crystals which hinder the exchange of cytokines in body fluids was confirmed from the X-ray powder diffraction results. Five characteristic peaks (peaks at 22.861°, 31.786°, and 34.054°) of the hydroxyapatite are clearly observed and conformed to the characteristic peaks of standard card No. Here, only a small amount of crystals will be generated, even after the B-BGs are sintered at high temperature (Figure 1G). As illustrated in Figures 1E,F, the weakening of the compressive strength and stress-strain curve of the B-BGs compared with that of HAs shown the important role of microstructures on mechanical properties. In this study, the compressive strength of B-BG was ~1.2 MPa, much lower than 4.0 MPa of the hydroxyapatite scaffold. As shown in Figure 1H, after soaked in SBF, the B-BGs exhibited many additional new XRD signals, which are the characteristic peaks hydroxyapatite. Which confirmed the *in vitro* mineralization experiments indicated that after 42 days of immersion, most of the undegraded B-BGs became newly generated hydroxyapatite. Additionally, the inert materials that are hard to degrade can cause secondary fractures because of the unmatched strength of these materials and that of the new bone. During the movement of the mandible, the implanted scaffold wears out forming free particles, which cause embolism or inflammation (Koff et al., 2019; Qiu et al., 2020). However, the biodegradable glass can circumvent this problem.

### *In vitro* Cell Compatibility

**Figure 2:**
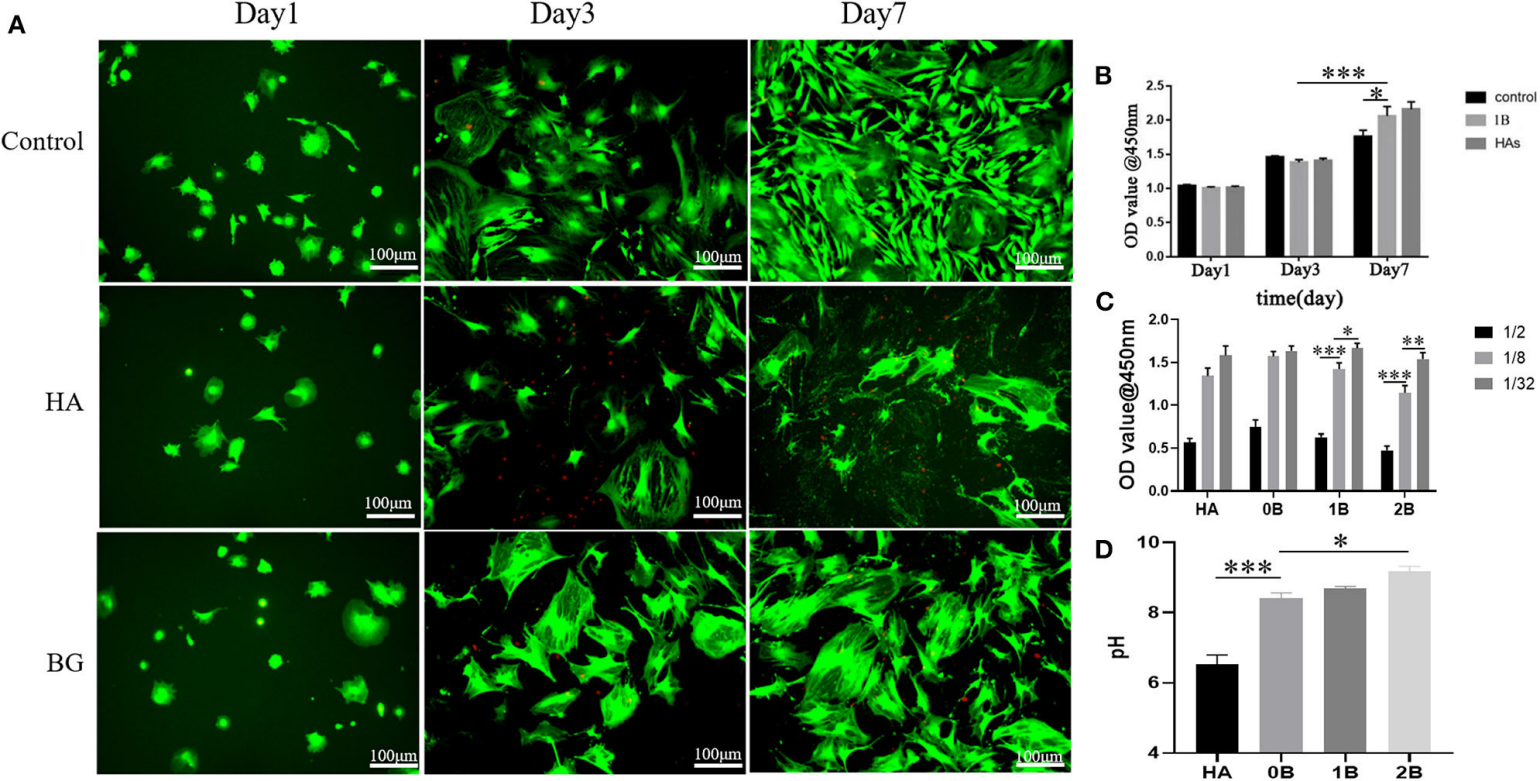
*In vitro* cytotoxicity evaluation of BMSCs using the CCK8 assay and the Live/dead cell staining technique. **(A)** Live/dead cell staining for BMSCs with treatment of media (control), has an ionic dissolution product (HA), and 1B-BGs ionic dissolution product (BG); **(B)** Cell survival rate at different exposure time using different treatments; **(C)** Cell survival rate at different concentration of ionic dissolution products; **(D)** the pH value of different ionic dissolution products. *P < 0.05, **P < 0.01, and ***P < 0.001.

Since it is the first step in the process of bone reconstruction, this study further analyzed the fate of mesenchymal cells-osteoblasts which is crucial in repairing defective bones. Here, we identified that the optimal pH for the proliferation of osteoblasts was 8.4 (Figure 2C), and that it is a major mechanism by which the bioglass scaffold promotes bone repair (Galow et al., 2017; Zamani et al., 2019). The long-term cytotoxicity of scaffolds from the BMSC were measured using the CCK-8 assay. These cells were treated with increasing concentrations of the scaffold ionic dissolution product at days 1, 3, and 7. Although there was no significant difference in all these days compared to the blank group, the alkaline environment which was above the stated physiological pH did not cause great damage to these cells. As illustrated in Figure 2, the B-BGs exhibited a certain level of toxicity in the high-concentration of ionic dissolution products, but still, they have a higher biocompatibility than hydroxyapatite (Figures 2B,C). To simulate the fate of the cells in close contact with the scaffold, a scaffold ionic dissolution product ratio of 1: 8 was used. Then, cell viability was tested using either live or dead cell staining techniques (Figure 2A). On the first day, the cells showed a round shape instead of the fusiform shape that is common to many mesenchymal stem cells, and this could be attributed to the fact that the plated cells did not fully extend on their first day of culture. However, from the normal spindle cells, flat and large cells appeared after 3 days of culture and this could be due to active osteoblasts, differentiated cohort cells, epithelial cell contamination, or the intolerance of the epithelial cells to alkali. Therefore, as illustrated in Figure 1A, this study affirms the first two speculations.

### *In vivo* Osteogenesis of the Scaffold

**Figure 3:**
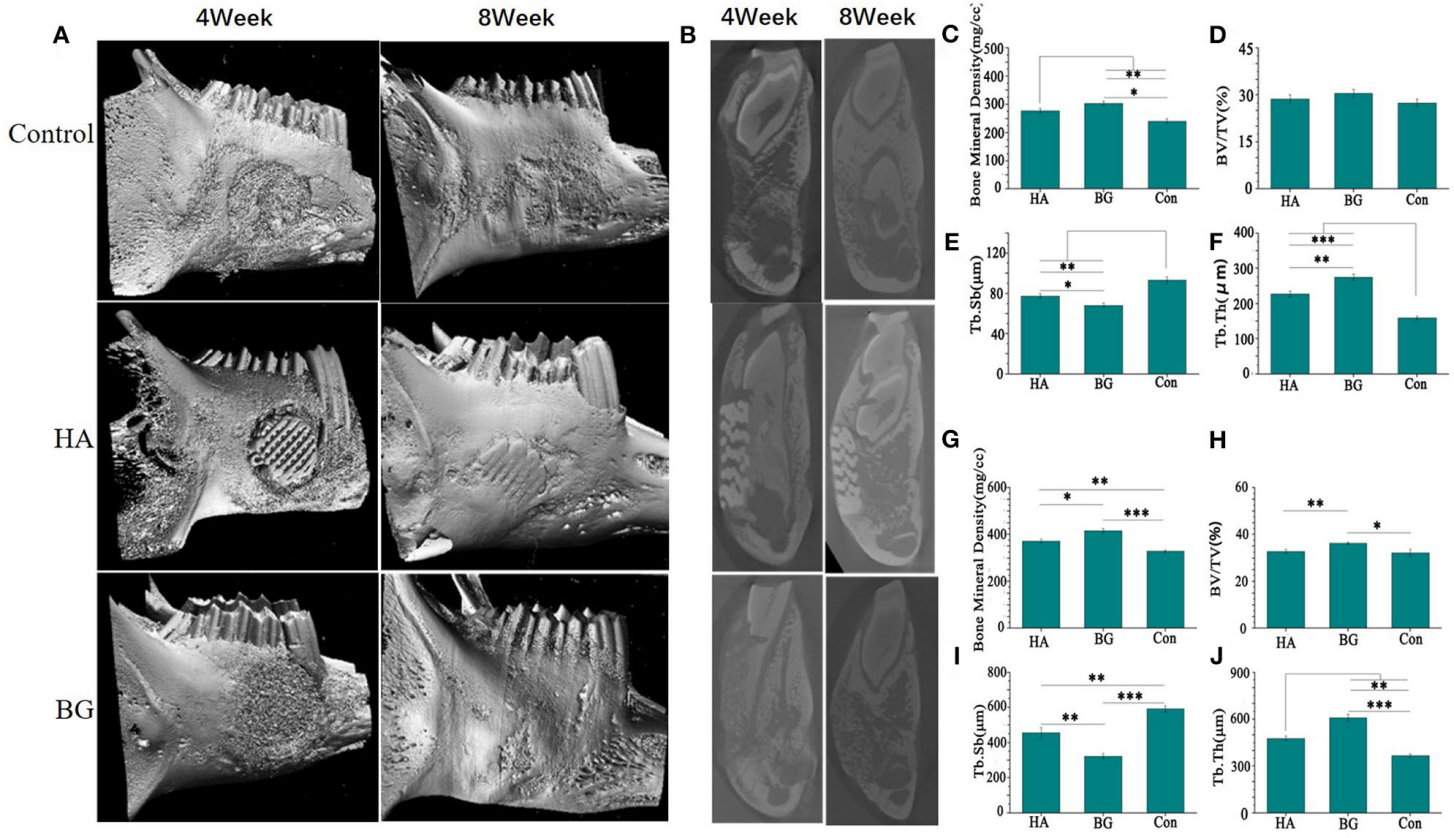
The CT scan image**(A)** and sagittal image **(B)** of the harvested mandible healed for 4 weeks and 8 weeks; The bone mineral density (BMD) after 4 weeks **(C)** and 8 weeks **(G)**; The bone volume density (BV/TV) after 4 weeks **(D)** and 8 weeks **(H)**; The trabecular separation, (Tb.Sp) after 4 weeks **(E)** and 8 weeks **(I)**; The trabecular thickness(Tb.Th) after 4 weeks **(F)** and 8 weeks **(J)**. *P < 0.05, **P < 0.01, and ***P < 0.001.

**Figure 4:**
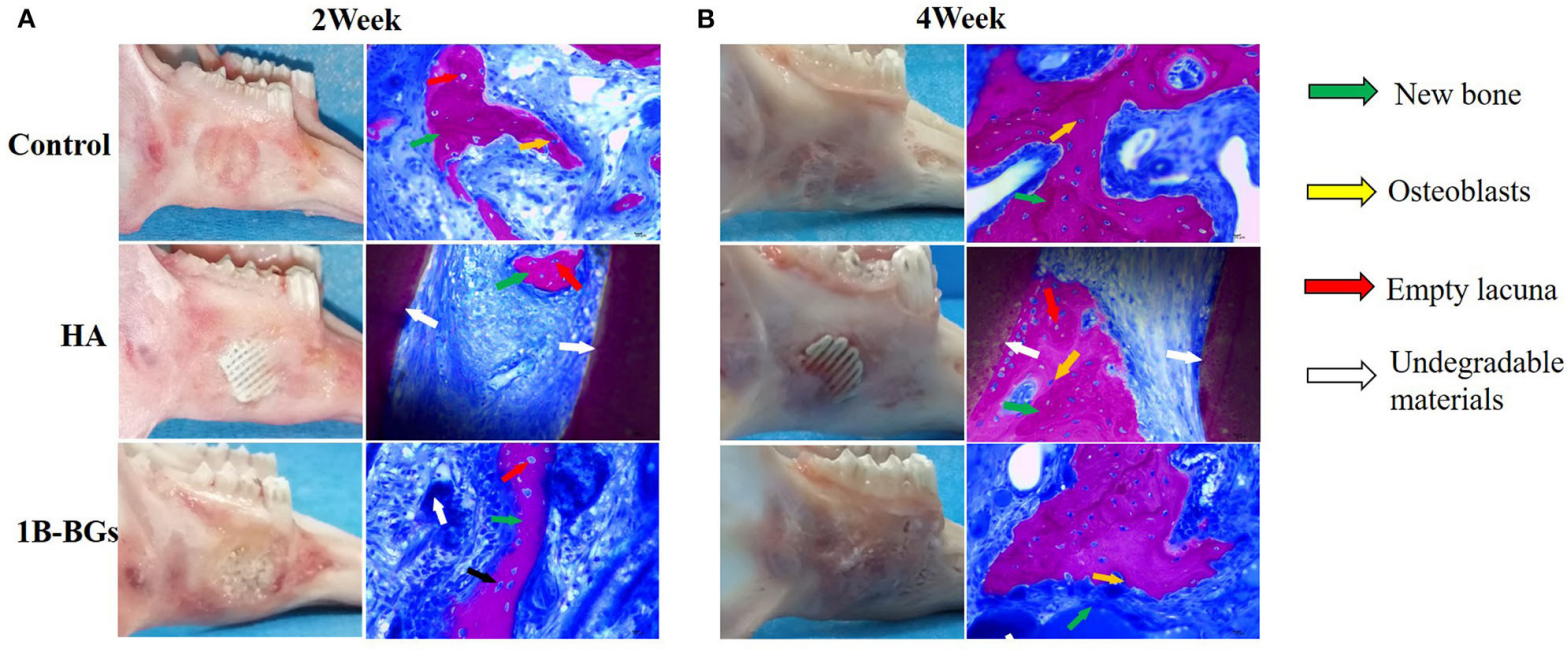
Lower jaw tissue harvested after 2 and 4 weeks of treatment. Image of lower jaw and HE staining pictures after **(A)** 2 weeks and **(B)** 4 weeks of treatment. The osteoblasts (yellow arrows), new bones (green arrows), empty bone pockets (red arrows), and undegraded material (white arrow) were marked.

The most direct evidence to evaluate the ability of the scaffold in promoting bone repair is through direct osteogenesis conducted in animals. This study exemplifies that small-animal models presents several challenges and limitations during the reconstruction of the mandible. Furthermore, we performed *in vivo* experiments to test the strength of the mandibles that were repaired using the bioglass implant. As illustrated in Figure 1E, our study established that despite the low strength of the boron-containing bioglass scaffold, all the experimental animals could eat on their own within 3 days after surgery. This shows that the bioglass scaffold can still perform certain functions under specific size defects in place of the defected bone. CT scans and *in vitro* jaw images show that the B-BGs have the fastest ability for bone repair mechanism. Here, results also showed that in the second week, it had degraded and repaired most of the defect structure. **Figure 4** shows that in the fourth week of the defect image, the defect repaired using the 1B scaffold had the most complete and smooth structure. The quantitative CT scan results are illustrated in Figure 3 which shows that the B-BG-repaired defects have the following characteristics of the bone; highest density, volume fraction, and trabecular thickness compared to both the hydroxyapatite and the blank control groups. This indicated that the B-BGs produced the new bone mass during the repair process (Figures 3A–D,F–H,J). In all the above cases, we observed mild oozing from the oral incision site, perhaps due to the underlying mild inflammation that occurred 2 weeks after surgery. Nevertheless, this was completely resolved through the 4-weeks recheck examination. A short-term BMP-induced inflammation is commonly reported and it begins on the 3rd day after surgery, peaks at 1 week, and typically resolves 2–3 weeks postoperatively (Lee et al., 2012). This response is anticipated as the bioglass scaffold exhibits chemotactic activity for inflammatory cells such as mononuclear and poly-morphonuclear cells and osteoclast-like cells. From our study, we, therefore, conclude that the general dose and method of application that uses the bioglass scaffold is viable, despite the minimal inflammation and mild oozing, hence, it is clinically appropriate as it resolves defects spontaneously by the 4th week after surgery.

The CT scan image formed a more complete bone tissue result. The boron-containing bioglass scaffold has the lowest trabecular bone separation. This means that the new one has the strongest strength and can efficiently treat the mandibular defects caused by bone resorption or other systemic diseases (Figures 3E,I). The methylene blue/magenta staining results further confirmed the CT scan results. Here, the B-BGs had the most osteoblasts (yellow arrows), more new bones (green arrows), and the least empty bone pockets (red arrows), nevertheless it is also observed in slices even in the fourth week after surgery. However, they partially contained undegraded material (white arrow). This could be due to the deficiencies of the sol-gel method used in the preparation of bioglass itself, that is, it is easy for insoluble calcium salts and silicates to be produced during the preparation process of bioglass scaffolds (Figure 4).

When deciding on the best facial reconstructive procedures, it is important to remember that the ultimate goals of mandibular reconstruction are to restore speech, masticatory function, swallowing, respiration, and to preserve facial features. In this study, we prepared a boron-containing bioglass using the sol-gel method and used Sodium alginate as a solvent to prepare a bone defect repair bracket that can be quickly printed. Notably, when this technique is combined with CT three-dimensional reconstruction technology, it is likely to quickly customize repair brackets with special sizes and structures according to the patient defects through 3D printing. Through the boron-substituted bioglass scaffolds, we found bioglass with a higher and faster degradation rate, and can slowly mineralize in body fluids to promote bone reconstruction. Besides, it has a certain strength that supports the normal functioning of the defective mandible. Also, it has good bone induction ability and can quickly repair damaged mandibles. Moreover, it promotes bone reconstruction and slows down its resorption, so that the new bone has a higher strength. In future work, we can improve the strength of the biological glass scaffold so that it can be applied to larger defects.

## Conclusion

In this study, borosilicate bio-glass scaffolds matched with the defects were prepared by using CT scanning data combined with 3D printing technology according to the shape and size of the mandibular defects. we have demonstrated that 3D of printed boron-containing bioactive glass scaffolds have good biocompatibility. their pore structure provides transport channels for nutrients and metabolites for cell growth, facilitates cell proliferation and promotes bone regeneration. almost completely degraded within 4 weeks after transplantation, with the gradual degradation of materials, new tissues adapted to the morphology and function of their own bone tissue are formed to repair the defective tissue. All in all, B bio-glass provides new hope for the repair of mandibular defects.

## Data Availability Statement

The raw data supporting the conclusions of this article will be made available by the authors, without undue reservation.

## Ethics Statement

The animal study was reviewed and approved by Ethical Committee of Laboratory Animal Science Department, Nanchang University.

## Author Contributions

PZ, KY, and LL contributed to conception and design, data acquisition, analysis, interpretation, and drafting of the manuscript. ZZ and XZ contributed to data acquisition and drafting of the manuscript. WL, CC, and KZ contributed to interpretation and critical revision of the manuscript. FA contributed to conception and design, interpretation, and critical revision of the manuscript. All authors gave final approval and agreed to be accountable for all aspects of the work.

## Conflict of Interest

The authors declare that the research was conducted in the absence of any commercial or financial relationships that could be construed as a potential conflict of interest.
